# Bibliographical Notices

**Published:** 1849-06

**Authors:** 


### BIBLIOGRAPHICAL NOTICES.

*Practical Pharmacy; the Arrangements and Manipulations of the Pharmaceutical Shop and Laboratory.* By FRANCIS MOHR, Ph. D., Assessor Pharmaciæ of the Royal Prussian College of Medicine, Coblentz; and THEOPHILUS REDWOOD, Professor of Chemistry and Pharmacy to the Pharmaceutical Society of Great Britain. Edited, with extensive additions, by WILLIAM PROCTER, Jr., Professor of Pharmacy in the Philadelphia College of Pharmacy. Philadelphia: Lea & Blanchard, 1849.

The class of technical works designed to illustrate the practical operations necessary in the various arts, long popular with the Germans as Hand-books, and with the French as Manuals, has of late years become common amongst us, and although skill and readiness in any art can only be thoroughly acquired by practice, there is no doubt but that their acquisition may be much facilitated by properly adapted book instruction.

Of this class is the work now before us. It is a treatise on Practical Pharmacy, embracing a description of the manipulations and apparatus necessary in this art, and appears to fill all the requisites for a work of this character. It is full and minute without unnecessary diffusion, and contains instruction precisely of that practical kind which is to be desired in a manual of pharmaceutical operations, and which is not to be found in works of more scientific pretensions.

The reputation of Mohr and Redwood, the original authors, is a guarantee that the work contains the results of the best practical experience of Germany and England, and the emendations and additions of Professor Procter in adapting it to the present state of American Pharmacy, are judicious and valuable. The emendations consist in the transposition and classification of Mohr and Redwood's matter, according to the subjects treated of, making it more convenient for reference, and neater and more systematic in arrangement; the addition of much valuable new matter which has increased the book more than one fourth in size, including about 100 wood cuts.

The work, as now published, contains about 570 large octavo pages, handsomely printed from good clear type, and illustrated by over 500 well executed wood cuts of chemical and pharmaceutical apparatus.

The First Chapter treats of the general arrangement of the shop or dispensary, the laboratory, the store-room, &c., and is of interest to apothecaries and such physicians in country localities as are under the necessity of preparing and dispensing themselves the medicines required in their practice. The Second Chapter contains minute directions as to weighing and measuring, the proper construction of balances, the various methods of determining the specific gravities of solids and liquids, hydrometric tables, &c. Two chapters follow on the construction of furnaces, the adaptation of fuel, and the various methods of applying heat, the effect of temperature in pharmaceutical operations, &c. After which are described the mechanical contrivances for the comminution and pulverization of drugs, such as mortars, cutting knives, drug mills, &c. The method of separating precipitates, use of filters, the clarification of liquids, and decolorization of syrups and oils by the use of charcoal, are very fully described, as are also the various forms of hydraulic and other presses for pharmaceutic uses.

In the Ninth Chapter, which treats of the methods of obtaining solutions for syrups, extracts, tinctures, &c., will be found a very useful table of solubilities which has been incorporated with the work by the American editor, taken chiefly from Henry and Gibourt. In this connection the displacement process is elaborately explained and examined, and the conclusion the authors arrive at coincides with our own long since formed, that except in circumstances where expedition is especially important, as, for instance, the preparation of small quantities of tinctures or infusions for immediate employment, the old method of maceration or digestion, with occasional agitation and subsequent expression of the liquor from the mark or dregs, is to be preferred.

The various precautions necessary in the preparation of extracts are carefully noted, and all the different forms of evaporating apparatus well described and figured. We extract the following in reference to a class of extracts little known.

"There are some drugs which contain so large a proportion of volatile oil and resin, that these may be extracted as fluid or semifluid, oleo-resins, analogous to copaiba, and highly eligible for medicinal use, owing to their uniform strength and consistence. Cubebs, black pepper, capsicum, ginger, cardamom seeds, pellitory, male fern, and wormseed are among these substances. The menstruum employed is ether. The drugs in moderately fine powder are compressed in a displacer (fig. 270,) and treated directly with the ether, until as much tincture is obtained as equals twice the weight of the substance treated. When cubebs are thus treated, the ethereal tincture is introduced into a retort, and by means of a water bath kept at 120° Fahr., seven-eighths of the ether is distilled off, observing to introduce some fragments of glass into the retort to facilitate the liberation of the ethereal vapour. The fluid residue is then placed in an evaporating capsule, and left exposed until the remainder of the ether passes off. Cubebs yield about 15 per cent. of a fluid oleo-resinous extract by this process. In the case of cardamoms, the extract consists of volatile oil, fixed oil, and a little resin, and is so entirely fluid and permanent, that it may be used with great propriety in pharmacy, for aromatizing powders in prescriptions. The presence of the fixed oils tends to preserve the volatile oil, which, when pure, soon changes by the exposure to the air."—p. 310.

The ethereal extract, or oleo-resin of cubebs, we look upon as a valuable preparation. It has been much used in the practice of one or two physicians of this city, with satisfactory results. Ten or twelve drops fully represent the medicinal activity of one drachm of cubebs in powder, and the diminished bulk makes it much more eligible for administration. It may be given dropped on sugar, or floating on a tablespoonful of water, or made into a pill mass by the admixture of an equal weight of magnesia. The essential oil of cubebs is frequently prescribed under the impression that it is the active principle. This is an error; the peculiar sensible and medicinal properties of the piperaceæ do not reside in the essential oil, but chiefly in the soft resin. Thus we find the essential oil of the piper nigrum almost tasteless, the ethereal extract or oleo-resin, on the contrary, sometimes, but improperly, called the "oil of black pepper," is powerfully acrid.

The following refers to a class of preparations but little noticed in this country, which we think country physicians might frequently cause to be prepared from indigenous vegetables for their own use:

"Preserved Juices, or Alcoolatures.—M. Beral, some years since, proposed a class of tinctures of narcotic plants, in which the waters of vegetation of recent juices acted as the water of dilution of the alcohol. There are two ways of proceeding—one by adding alcohol to the bruised plant, macerating and expressing;—the other is to express the juice, and add sufficient alcohol to it to preserve it. The latter is the plan of Mr. Squire, of London, who, under the name of preserved juices, introduced these tinctures to the attention of English pharmaceutists. The juice obtained at the time most favourable to the development of the virtues of the plants, is mixed with half its weight of alcohol, and after a time filtered. Wormwood, taraxacum, and the narcotic plants are thus treated. The advantage proposed to be gained by these preparations is, that the active constituents of the plants are unchanged by the process of drying. Owing to the variable proportions of water in the juices of the same plant, at different times, they are liable to vary in strength from this cause. Mr. Redwood found that the juice of the Lactuca sativa had the sp. gr. 1.031 in the evening, and 1.026 the following morning." p. 279.

The distillation of essential oils, &c., the rectification of ether and alcohol, and the sublimation of benzoic acid, calomel, &c., are fully described, as are also neutralization and the use of test papers with minute directions in acidimetry and alkalimetry. We quote the following observations on the interesting subject of the extraction of certain essential oils, which do not exist ready formed in the substances from which they are obtained, but are formed by reaction between their proximate constituents, during the process to which they are subjected.

"There are a few substances in obtaining essential oil, from which it is necessary to conduct the process of distillation in a peculiar manner. Bitter almonds, mustard seeds, and horseradish, are of this class. These substances do not contain essential oil ready formed, but the oil is produced in the process to which they are submitted. Bitter almond cake, for instance, from which essential oil and a distilled water are obtained, contains two bodies, amygdaline and emulsine, by the mutual reaction of which, together with water, the oil is produced. Black mustard seed, also, contains two analogous bodies, myronic acid and myrosyne, which, in like manner produce the essential oil of mustard. Bodies of analogous character exist in horseradish and in laurel leaves. The emulsine and myrosyne of the almond and mustard seed, and the corresponding substance in the other plants mentioned, undergo a change similar to the coagulation of albumen, when heated to a temperature approaching that of boiling water, and when thus coagulated they are no longer capable of producing essential oil with the other vegetable principles. It is necessary, therefore, in obtaining essential oil from this class of substances, to macerate them with cold or lukewarm water, for some time previous to the application of heat. If boiling water were added at once to these substances, it would coagulate and render inoperative one of the constituents from which under other circumstances, the oil would be formed. In distilling bitter almonds, the cake, which has been previously freed from fixed oil by expression, is powdered and mixed with about twenty parts of cold water. The mixture is allowed to stand for twenty-four hours before the application of heat, during which time the essential oil is produced; it is then submitted to distillation." p. 353.

The 13th chapter treats of the absorption of gases, in connexion with which is given the method of preparing Quevenne's metallic iron reduced by hydrogen.

Chapter 14th is entirely new, being the work of the American editor. It is a valuable treatise on the preparation and purification of the fixed oils and fats used in pharmacy, and on cerates, ointments, soaps and plasters. From this chapter we extract the following:

*Fixed oils as obtained by expression.*—"The oil as it runs from the press is rarely fit for use. It has in the case of castor oil, albuminous and watery particles admixed, and requires to be boiled with water, during which the albumen is coagulated, rises to the surface and assists in the clarification of the oil. This is skimmed off, the oil ladled from off the surface of the water after boiling, and is subsequently heated to remove the watery particles which adhere to it. It is in this last operation that the oil is most liable to be injured, because as soon as the water is vaporized, the temperature of the oil rapidly rises, and it acquires acrimony in proportion. If steam heat, under a pressure of one or two pounds per inch, were employed, no such injury would result.

Fixed oils are generally coloured, though not so naturally. The yellow hue of the oil of almonds is due to the colouring matter of the episperm of the kernel, and the yellow dust which adheres to it, due to attrition. The separation of these by blanching would be too tedious, although sometimes done for particular uses.

"The second process by boiling the bruised seeds in water after having gently torrefied them, and skimming off the oil as it rises to the surface, was formerly adopted in making castor oil; and much of that now used for domestic purposes in the West Indies is thus made by the negroes. Oil thus prepared is greatly inferior to that extracted by expression. This second process is resorted to in the extraction of cod-liver oil. The livers freed from the membranes are cut in pieces, placed in a tin vessel with a portion of water, and heated. The oil gradually rises to the surface and is skimmed off, to be subsequently clarified by straining, &c. The more recent the livers, and brief and moderate the heating process, the lighter coloured and less offensive the oil. The more common variety is obtained by throwing the livers in heaps, exposed to the sun, during which they undergo decomposition, and the oil, dark coloured and rancid, flows from them to a suitably arranged receptacle. According to M. Jongh, the light-coloured, carefully prepared oil, contains more iodine and bromine than the dark rancid variety."

"Lard, the adipose matter of the hog, varies much in value for pharmaceutical purposes, as found in commerce. This arises chiefly from the frequent want of care in its preparation, and partially to the part from which the lard is obtained. The fatty deposit of the omentum, mesentery, and in the region of the kidneys, is that most appropriate for pharmaceutic use, because it requires less heat in rendering it, and the lard has a smoother and more homogeneous texture. So important is good lard in the fabrication of some cerates and ointments, that when it is not attainable of unexceptionable quality, it is worth while for the apothecary to render it himself from selected fat. This should be cut in small pieces, freed as much as possible from the adhering membranes, introduced into a boiler with a portion of water, and boiled until the fat is fused from the cellular tissue, in which it is naturally deposited, and the water has all evaporated. As soon as this is the case, and before the temperature of the fat rises much above 212°, it should be dipped out and strained. It is the excessive heat employed at this point of the process, together with its influence on the pieces of flesh and membranes which generally accompany the crude fat, that the inferior quality of much of the commercial lard is attributable."

When lard or other fats are rendered in this way it is difficult to free them entirely from adherent water, the presence of which tends to produce rancidity by promoting oxidation. We prefer the method generally adopted by perfumers in preparing them, which is, after slicing the crude fat, to beat and triturate it in a marble mortar until the cellular tissue is sufficiently torn to allow the exudation of the fat on melting, which should be done in a porcelain vessel by the heat of a water or salt water bath, and the fat, when sufficiently liquified, separated from the membrane by straining through a double flannel bag without expression. Thus prepared it can be preserved for a long period perfectly free from rancidity if excluded from the air.

The remainder of the book with the exception of the last chapter is occupied with useful practical observations regarding various miscellaneous operations, such as sealing and bending glass tubes, connecting and luting apparatus, covering the outside of glass flasks, &c., with a protecting coat of copper by galvanic deposition, the method of preparing gelatine capsules for the administration of copaiba, &c., graduating measures, the various contrivances for the inhalation of ether and chloroform as anæsthetic agents, &c., and on the various manipulations requisite in extemporaneous pharmacy, well calculated to instruct the operator in the pharmaceutical shop and laboratory.

The final chapter may be regarded as an appendix, treating of the use of chemical reagents and the method of ascertaining their purity. This chapter has been added to the original work by the American editor, and will be found a convenient compend of the matters referred to.

It is difficult to convey a satisfactory idea of a work of this kind, treating as it does, of many various subjects in the limited space at our disposal, and we are conscious that in this hasty notice we have given a very inadequate impression of its merits. It is a book, however, which we suppose will be in the hands of almost every one, who is much interested in pharmaceutical operations, as we know of no other publication so well calculated to fill a void long felt in the absence of a practical work of this character.

In conclusion we would again allude to the excellent mechanical execution of the work, which is very creditable to the taste and liberality of the enterprising publishers.

---

*Chemical Analysis, Qualitative and Quantitative.* By Henry M. Noad, Lecturer on Chemistry at St. George's Hospital, etc., etc. With numerous additions by Campbell Morfit, Practical and Analytic Chemist, &c. With illustrations. Philadelphia. Lindsay & Blakiston, 1849. pp. 572.

Perhaps there is no species of knowledge that exhibits the intellectual advancement of modern times more strikingly, than that by which the elements of a compound, natural or artificial, may be separated and their quantities determined, often with the greatest accuracy. Rare and valuable substances, as lapis lazuli, highly prized in the arts, have been successfully imitated by resorting to this scientific "open sesame," and now, by extending its principles to the organic productions, bodies hitherto only elaborated by the mysterious process of secretion, have been created by the chemist after the agency of analysis has pointed out their elementary constitution. In the United States the cultivation of chemical science has not kept pace with some other departments, and has been more directed to the mineral kingdom in connection with geological pursuits, than to extending the boundaries of the science itself. This has arisen partly from the sparsity of practical schools, and the scarcity of treatises on analysis. The last deficiency is being rapidly done away, as we already have more than one manual, and the book under the above title is now presented as a new and valuable offering to the student.

The work of Mr. Noad consists of two parts, qualitative and quantitative analysis. After describing the reagents usually employed with directions for ascertaining their purity, our author notices the reactions which mark solutions of the simple bases and acids when brought in contact with these reagents, as well as the evidence afforded by the blow-pipe, forming a set of maxims or characters by which each substance may be recognized in a separate state. The following chapter is devoted to the explanation of systematic qualitative analysis, in which substances are recognized in the presence of each other, due allowance being made for their mutual influence on the tests. This knowledge requires a very considerable degree of memory and close familiarity with reagents to give proficiency, and has been carefully and minutely explained in the pages before us. Examples are given of the modes of procedure in identifying mixed bases, and mixed acids, and the poisonous acids and metals have received a special notice. This part of the work is terminated with tables illustrating the several steps of the processes for determining the nature of bases and acids.

The second part of the book enters fully into the details of quantitative analysis, commencing with the alkaline metals. It need hardly be said that the author has availed himself of the standard works of Rose and others, in the composition of his task, as well as the mere recent facts and suggestions interspersed through the Journals. The immense importance of this branch of chemistry to the progress of the arts, and to the science of chemistry itself, places it in the foremost rank of chemical studies. It is that which has received the smallest share of attention in this country, and it is to be desired that the facilities for its cultivation afforded by practical schools, will be increased and patronized. In no country is there a greater field for the application of chemical knowledge to the benefit of society, by increasing its resources in the several regards of agriculture, arts, and mines, than in this; and to the organic chemist the numerous items of materia medica which originate from our wide spread soil, opens a field for the exercise of his favourite pursuits, teeming with objects of interest.

Our author has devoted about forty pages to the method of ultimate organic analysis. American chemists have hardly as yet touched this department of analytical investigation, and the accuracy of the instruments, and the expertness of the manipulation required to give value to its results, may afford a good reason in a country where as yet the patrons of science are nearly all found in the ranks of art and manufacture, whilst but comparatively few are prepared by acquirements or means, to tread the quiet, unobtrusive and unprofitable pathway of abstract scientific pursuits.

There is one branch, however, that has not been treated of as a distinct subject by our author, or by any other writer on analysis with whom we are acquainted, and that is *organic proximate analysis.* There are scattered through the journals and standard works a large number of facts, that if brought together in the form of a special treatise on proximate analysis, would prove extremely useful to a large class of chemical inquirers. We are well aware of the difficulties of the subject—that in many cases the bodies to be isolated are very complex and readily undergo change in the process—yet we believe that sufficient observations exist to form the ground work of a valuable manual. In this, as in inorganic analysis, two departments would be required: the detection of each constituent, and the determination of the proportion of each ingredient. This last is probably the next difficult problem in analytical investigations, and the wide differences that are presented in the results of even distinguished operators, is a sufficient evidence of the reality of this opinion. The importance of this kind of analysis is becoming daily more important in reference to pathology, physiology and pharmacy. The composition of drugs and the relation of their active constituents to the mass of associated matter, is too important in the construction of formulæ and their practical accomplishment, to need comment, and we trust that some well qualified individual among the numerous organic chemists abroad, will fill this hiatus in chemical literature.

"The care and fidelity with which the author has performed his laborious task, has left little more to be done by the editor than to make such additions as are called for by the latest investigations in chemical analysis." We observe, however, that the introductory remarks in the original on manipulation have been omitted as too succinct "to be serviceable to the student," who is referred to a larger work for this information, whilst a chapter on equivalent proportions, chemical notation, and the principles of nomenclature have been substituted. Now we have not the slightest objection to the introduction of this chapter, which adds to the usefulness of the book, nor to the reference to the editor's work on manipulation, but we decidedly protest against the omission of the author's remarks, merely on the ground that they are not sufficiently extended; for although we agree with the editor that they are too brief for a thorough teaching, yet they contain valuable hints that were altogether in place at the commencement of such a work as that under consideration.

---

*A Manual of Auscultation and Percussion.* By MM. Barth & Roger. *Translated with additions, by* Francis G. Smith, M. D. Philadelphia. Lindsay & Blakiston, 1849. Second edition.

This little work contains an exposition of the more important physical signs of disease. It is principally taken up with a description of those derived from the thoracic viscera, but includes, besides, a sufficiently full account of the application of auscultation and percussion to the recognition of the morbid states of other organs of the body. We are first made acquainted with the proper method of conducting physical exploration, and with the signs afforded through these means in the *healthy* state of the organs. As the respiratory apparatus responds to our inquiries more fully and distinctly than any other, to it is given the first place, and the alterations in its physiological condition classed and described according to their grade, rythm, character, and the presence of abnormal sounds. These signs are then presented to us in a tabular form, (as arranged by Dr. Walshe,) by which, at a glance may be seen their physical cause, ordinary seat, and the diseases in which each one is observed. The physical signs afforded by the circulatory apparatus, and which are appreciable by auscultation, are next described in the same manner, after which follows a concise enumeration of the signs furnished by auscultation of the abdomen, head, extremities, uterus, and fœtal heart. Part II. commences with a short historical notice of percussion, followed by an account of the application of this method of exploration, treated in the same order as that by auscultation. We notice that the translator has appended a description of the employment of both these methods combined, as introduced into practice by Drs. Camman and Clarke. On the whole, there is no better nor more succinct account of this important branch of medical diagnosis than the manual of MM. Barth and Roger. The arrangement of its contents is natural and lucid, and the reasoning of the authors is marked by a mathematical conciseness and precision, which enable the reader to follow their analyses with the most perfect satisfaction. It has been translated into various modern European languages, and the fact of Dr. Smith's translation having already reached a second edition, sufficiently proves that is justly appreciated in our own country.

---

*A Practical Compendium of Midwifery; being the Course of Lectures on Midwifery and on the Diseases of Women and Infants, delivered at St. Bartholomew Hospital, by the late* Robert Gooch, M. D. Prepared for publication by George Skinner, Member of the Royal College of Surgeons, London. Fourth American Edition. Philadelphia, Barrington and Haswell, 1849.

The above Volume contains the substance of a Course of Lectures on Midwifery, delivered at St. Bartholomew Hospital by the late Dr. Gooch. As an effective writer, as a profound thinker, and as an experienced obstetrician, Dr. Gooch has no superior and but few equals, and it is a source of regret, that his brief career prevented him from publishing a more full and extended treatise upon Midwifery and the Diseases of Women. We do not mean by this remark to depreciate the value of the volume before us, for we know of no medical work, which contains, in an equal number of pages, so much that is practically useful to the obstetrical practitioner. Throughout its pages, the great principles of obstetrical science are propounded in a style peculiarly concise and wonderfully calculated to leave a lasting impression on the mind of the reader.

In the first lecture, we have an account of "the natural functions, and of the diseases of the female organs of generation." Our author's remarks on Chlorosis, Amenorrhœa, Dysmenorrhœa, and Menorrhagia are admirable, containing in our opinion, in a few words, all that is required for a proper comprehension of the pathology and treatment of these diseases.

The second lecture contains a pretty good account of Conception—of the Gravid Uterus—of the signs and diseases of Pregnancy, &c. The article on Abortion is most excellent, and we would recommend its careful perusal to all who may wish to comprehend the best mode of managing this unpleasant and sometimes dangerous accident.

Lecture 3d is devoted to the consideration of Labour—the phenomena of which are well described. The major portion of this chapter contains some capital remarks on the delivery of the placenta. The skilful management of the placenta involves duties of the highest responsibility, and our author very justly remarks that "all anxiety with the family ceases, as soon as the child is born; but mine there begins: and if the friends of the patient knew as well as I do, the danger liable to attend the separation of the placenta, they would feel as I do." Thus impressed with the difficult duty of the obstetrician, we need not wonder that Dr. Gooch has devoted many pages to its consideration—leaving behind him a model chapter on the best and safest method of managing the delivery of the after-birth, with which we are acquainted.

Difficult labours are treated of in the fourth lecture. These deviations from natural labour are arranged under three heads: 1st, Impeded labour; 2d, Unnatural presentations; 3d, Complicated labour.

In impeded labours, the position of the child is supposed to be natural; its delivery being interfered with, by undue size of the fœtus; by contractions of the pelvis; by rigidity of the soft parts; by debility, &c. &c. The mode of ascertaining the various causes of impeded labour, as well as the treatment requisite in each case are clearly pointed out by our author. In commenting upon those positions of the head, in which the occiput is placed posteriorly, our author remarks, that "the difficulty in these cases is overrated—if the face is towards the pubes, this position is certainly unfavorable to an easy delivery; but if this deviation is not complicated with other causes of impediment, it is of no great consequence. To accomplish the delivery may require a few additional pains, but not in general the use of instruments as is by some supposed." The import of these remarks is peculiarly practical, but our author does not seem to have been aware of the frequency of these positions, or of the constancy with which, by the natural efforts of the female, the occiput is rotated round towards the pubic arch. The more recent observations of M. Nægelè on this subject, have established the truth of these facts—with which, we think, the reader should be made acquainted in a subsequent edition of this work.

The remaining portion of this lecture is taken up with an account of the injuries incident to protracted labour, including a description of the mode in which the forceps should be applied, &c. &c.

Under the head of preternatural presentations, we have included those of the breech, arm and funis. The management of arm presentations is exceedingly well described, and we would refer the reader to our author's excellent remarks upon the best mode of turning the fœtus in utero.

Labours may be complicated by the occurrence of convulsions, and of hemorrhage—by plurality of children, by rupture of the uterus, &c. &c. The sections on Convulsions and Hemorrhage are full of practical wisdom—forming an invaluable guide in the management of these dangerous accidents.

Lecture 6th contains a history of the general management of women after delivery.

In Lecture 7th we find more than twenty pages devoted to the consideration of the causes, symptoms and treatment of Puerperal fever—of Inflammation of the Uterus—of Puerperal Mania—of Phlegmasia Dolens and of Inversion of the Uterus. The remarks on these interesting subjects, though concise, contain much valuable information to the physician engaged in active obstetrical practice.

Lecture 8th contains a succinct account of the general management of Infants—of their Congenital Malformations, and of the diseases to which they are liable.

In conclusion we would commend the publishers for the handsome style in which the work is gotten up, at the same time suggesting to them, the propriety of having a work of such intrinsic worth put into the hands of some capable Physician, for the purpose of adding such notes as may be necessary to bring it up with the existing state of obstetrical science.

